# Weakly Positive Anti-NMDAR Encephalitis Following a Gastrointestinal Prodrome: A Case Report

**DOI:** 10.7759/cureus.107368

**Published:** 2026-04-19

**Authors:** Maira Khalid, Rashid Naseem, Azhar Hussain, Aurang Zaib, Muzammil Nadeem

**Affiliations:** 1 Department of Internal Medicine, Liaquat College of Medicine and Dentistry, Karachi, PAK

**Keywords:** anti-nmdar encephalitis, autoimmune encephalitis, electroencephalography, encephalopathy, gastrointestinal prodrome, plasma exchange, seizures

## Abstract

Anti-NMDAR encephalitis is an autoimmune neurological disorder characterized by neuropsychiatric symptoms, seizures, and altered consciousness. We report a 35-year-old female who presented in March 2025 with gastrointestinal symptoms followed by encephalopathy and seizures. Investigations revealed lymphocytic cerebrospinal fluid (CSF), diffuse slowing on electroencephalography, and parieto-occipital cortical changes on MRI, with weakly positive anti-NMDAR antibodies. The patient showed significant clinical improvement following corticosteroids and plasma exchange. This case highlights the diagnostic challenge of autoimmune encephalitis presenting with gastrointestinal prodrome and elevated inflammatory markers mimicking infection.

## Introduction

Anti-NMDAR encephalitis is a common form of autoimmune encephalitis and an important cause of acute encephalopathy in young adults, particularly females [1,2]. It typically presents with neuropsychiatric symptoms, seizures, and altered levels of consciousness and is frequently misdiagnosed as infectious encephalitis or a primary psychiatric disorder in the early stages [3,4]. Early recognition is critical, as timely initiation of immunotherapy is associated with improved neurological outcomes [5]. This report describes a patient who developed neurological deterioration following a gastrointestinal prodrome, highlighting a diagnostic presentation that may delay consideration of autoimmune encephalitis in the acute setting. 

Anti-NMDAR encephalitis is often preceded by a prodromal phase characterized by nonspecific symptoms, including fever, headache, malaise, and gastrointestinal manifestations such as nausea, vomiting, and diarrhea. Gastrointestinal symptoms have been reported in a significant proportion of patients and may lead to initial misdiagnosis as an infectious illness [4]. Additionally, the clinical significance of weakly positive anti-NMDAR antibodies remains a diagnostic challenge and requires careful interpretation in the appropriate clinical context.

## Case presentation

A 35-year-old woman presented in March 2025 with fever, vomiting, and diarrhea for one week, followed by progressive confusion and two generalized tonic-clonic seizures. On arrival, her Glasgow Coma Scale (GCS) score was 9/15. She was febrile and tachycardic, with otherwise stable vital signs. Her vital signs on presentation are summarized in Table 1. Abdominal examination revealed a soft, non-tender abdomen with no guarding or rigidity. Bowel sounds were present. Neurological examination revealed a GCS score of 9/15 with no focal neurological deficits. Pupils were equal and reactive, deep tendon reflexes were preserved, and meningeal signs were absent.

**Table 1:** Vital signs recorded in the emergency department on arrival GCS: Glasgow Coma Scale

Parameter	Value	Unit	Normal range
Blood pressure	100/60	mmHg	90-120/60-80
Heart rate	117	Beats per minute	60-100
Temperature	38.9	°C	36.0-37.5
Oxygen saturation	98	% (room air)	>94
Respiratory rate	16	Breaths per minute	12-20
GCS	9/15	-	15
Random blood sugar	128	mg/dL	70-140

Initial laboratory evaluation revealed anemia, thrombocytopenia, elevated inflammatory markers, and a markedly raised procalcitonin level. Cerebrospinal fluid (CSF) analysis demonstrated lymphocytic predominance with mildly elevated protein and normal glucose levels. Infectious workup, including herpes simplex virus polymerase chain reaction, was negative. Laboratory investigations and CSF findings are detailed in Table 2. An autoimmune encephalitis panel revealed weakly positive anti-NMDAR antibodies, while other autoimmune markers were negative (Table 3).

**Table 2:** Laboratory investigations and CSF analysis on admission CSF: Cerebrospinal fluid

Test		Patient’s value	Reference range	Unit
Hematology	Hemoglobin	8.3	12-16 (female)	g/dL
	White blood cells	83,000	4,000-11,000	cells/µL
	Platelets	1,37,000	150,000-400,000	cells/µL
Coagulation profile	Prothrombin time (PT)	13.3	11-13.5	Seconds
	Activated partial thromboplastin time (aPTT)	29.2	25-35	Seconds
	International normalized ratio (INR)	0.13	0.8-1.2	—
Liver and renal function	Aspartate aminotransferase (AST) (serum glutamic-oxaloacetic transaminase (SGOT))	42	10-40	U/L
	Alkaline phosphatase (ALP)	149	44-147	U/L
	Total bilirubin	1.9	0.3-1.2	mg/dL
	Urea (blood urea nitrogen (BUN))	18	7-20	mg/dL
	Creatinine	1.0	0.6-1.3	mg/dL
Electrolytes	Sodium	138	135-145	mmol/L
	Potassium	4.1	3.5-5.0	mmol/L
	Chloride	99	96-106	mmol/L
Inflammatory markers	Creactive protein (CRP)	19	<5	mg/L
	Procalcitonin	17.9	<0.1	ng/mL
	Herpes simplex virus (HSV) PCR 1 and 2	Negative	Negative	-
CSF	Chloride	120	115-130	mEq/L
	Protein	57	15-45	mg/dL
	Neutrophils	25	0-3	%
	Lymphocytes	75	40-80	%
	Glucose	83	50-80	mg/dL
	Gram stain and culture	Negative	Negative	-

**Table 3:** Autoimmune and antibody panel results

Test	Result	Reference value
Anti-NMDAR antibody	Weakly positive	Negative
ASMA	Negative	Negative
AGPCA	Negative	Negative
Anti dsDNA	Negative	Negative
APLA	Negative	Negative
P ANCA	Negative	Negative
C ANCA	Negative	Negative

Electroencephalography demonstrated diffuse generalized slowing without epileptiform discharges, consistent with encephalopathy. Brain MRI revealed diffuse cerebral cortical swelling, more pronounced in the parieto-occipital regions, with hyperintensities on T2-weighted and fluid-attenuated inversion recovery (FLAIR) sequences, suggestive of encephalitis. No evidence of acute infarction, hemorrhage, or mass effect was noted. Imaging figures could not be included; however, findings are described based on the official radiology report.

The patient was initially managed with empirical antimicrobial therapy, which was discontinued after exclusion of infectious causes. She subsequently received high-dose intravenous corticosteroids followed by plasma exchange. The patient demonstrated gradual neurological improvement with resolution of seizures and improvement in consciousness. At discharge, she was alert, oriented, and able to perform activities of daily living independently, with no significant residual neurological deficits. On follow-up at four weeks, the patient remained clinically stable with no recurrence of seizures or cognitive impairment and had resumed normal daily activities.

## Discussion

Anti-NMDAR encephalitis is a well-recognized autoimmune encephalitis characterized by prominent neuropsychiatric manifestations; however, an initial prodromal phase consisting of nonspecific systemic symptoms is increasingly described. These prodromal features commonly include fever, headache, malaise, and gastrointestinal symptoms such as nausea, vomiting, and diarrhea, which may occur in a significant proportion of patients and can lead to initial misdiagnosis as an infectious illness [3,4]. In the present case, the early gastrointestinal presentation contributed to diagnostic uncertainty and delayed consideration of an autoimmune etiology.

A notable and diagnostically challenging feature in this patient was the presence of markedly elevated inflammatory markers, including leukocytosis and procalcitonin, which are typically associated with bacterial infections. This raised significant concern for an infectious central nervous system process. However, extensive infectious workup, including negative CSF cultures and viral PCR testing, did not support an infectious cause. It is important to recognize that elevated inflammatory markers may also be observed in systemic inflammatory states and are not entirely specific for bacterial infection, particularly in critically ill patients [6]. This overlap can complicate the differentiation between infectious and autoimmune encephalitis.

The presence of weakly positive anti-NMDAR antibodies further added complexity to the diagnostic process. While strong antibody positivity is generally considered diagnostic, weak positivity must be interpreted cautiously and in conjunction with clinical presentation, CSF findings, and electroencephalographic abnormalities. In this case, the combination of characteristic clinical features, lymphocytic predominance in CSF, diffuse slowing on electroencephalography, and significant clinical improvement following immunotherapy strongly supported the diagnosis of anti-NMDAR encephalitis.

Neuroimaging findings in anti-NMDAR encephalitis are frequently normal or nonspecific, particularly in early disease stages [5]. However, when present, abnormalities may include cortical or subcortical signal changes. In this patient, MRI demonstrated parieto-occipital cortical swelling with T2-weighted and FLAIR hyperintensities, consistent with an inflammatory process. Although these findings are not specific, they provide supportive evidence when interpreted in the appropriate clinical context.

Early initiation of immunotherapy, including corticosteroids and plasma exchange, has been associated with improved neurological outcomes and reduced long-term morbidity in patients with anti-NMDAR encephalitis [7,8]. The favorable clinical response observed in this patient further supports the diagnosis and underscores the importance of early recognition and treatment. This case underscores the diagnostic challenge posed by overlapping features of infectious and autoimmune encephalitis and highlights the importance of maintaining a high index of suspicion for autoimmune etiologies in patients presenting with atypical features, including gastrointestinal prodrome and elevated inflammatory markers.

## Conclusions

This case highlights an atypical presentation of anti-NMDAR encephalitis characterized by gastrointestinal prodrome and markedly elevated inflammatory markers, initially mimicking an infectious process. Weakly positive anti-NMDAR antibody results should not exclude the diagnosis when supported by the overall clinical picture, CSF findings, electroencephalography, and neuroimaging. Early recognition of autoimmune encephalitis is essential to avoid delays in treatment. Prompt initiation of immunotherapy may lead to significant neurological recovery and improved outcomes. This case emphasizes the importance of maintaining a high index of suspicion in patients presenting with unexplained encephalopathy after nonspecific systemic illness.
